# Etiology and Outcome of Acute Intestinal Obstruction: A Review of 367 Patients in Eastern India

**DOI:** 10.4103/1319-3767.70617

**Published:** 2010-10

**Authors:** Adhikari Souvik, Mohammed Zahid Hossein, Das Amitabha, Mitra Nilanjan, Ray Udipta

**Affiliations:** Departments of Surgery, Calcutta, India; 1 Surgical Gastroenterology Medical College, Calcutta, India

**Keywords:** Etiology, outcome, intestinal obstruction

## Abstract

**Background/Aim:**

The etiology of acute intestinal obstruction, which is one of the commonest surgical emergencies, varies between countries and has also changed over the decades. We aimed to provide a complete epidemiological description of acute intestinal obstruction in a tertiary care hospital in Eastern India.

**Materials and Methods:**

This was a retrospective study of patients admitted in our unit with a diagnosis of acute intestinal obstruction between the years 2005 and 2008 at Medical College, Calcutta. The study comprised of 367 patients.

**Results:**

Acute intestinal obstruction was the diagnosis in 9.87% of all patients admitted with males (75.20%) grossly outnumbering females. The commonest age group affected was 20-60 years. In our patients, the main cause of obstruction was obstructed hernia followed by malignancy with adhesions coming third. Intestinal tuberculosis was an important cause for obstruction in our patients comprising 14.17% of patients. Conservative management was advocated in 79 patients while the rest underwent surgery. Postoperative complications occurred in 95 patients and of these, 38 patients had a single complication and the rest, more than 1. The main complications were wound infection, basal atelectasis, burst abdomen and prolonged ileus. The mortality rate was 7.35% (27 patients). The highest mortality occurred in those with intestinal tuberculosis.

**Conclusion:**

This study demonstrates that the pattern of intestinal obstruction differs from the Western world with obstructed hernias being the most important cause and also emphasizes the fact that intestinal tuberculosis assumes a prominent role. It also highlights the necessity of using universal precautions because of the ever increasing number of HIV patients in those with intestinal obstruction.

Acute intestinal obstruction is one of the most common surgical emergencies. Etiology of acute intestinal obstruction varies among different countries and there has also been a change over decades. The aim of our study was to provide a complete epidemiological description of acute intestinal obstruction in a tertiary care hospital in Eastern India.

## PATIENTS AND METHODS

We did a retrospective study of patients admitted to our unit during the period 2005 to 2008 at Medical College, Calcutta, with a diagnosis of acute intestinal obstruction, both small bowel and large bowel. Patients who attended to the emergency department in our hospital were referred from districts and all of them had a 2-3 day history of acute intestinal obstruction. All patients including those subjected to non-operative intervention were closely reviewed in a place of close observation and a detailed analysis of the clinical notes of the junior doctors, surgeons, and nurses was made. Criteria for admission were obstipation (279 patients: 76.02%), colicky pain (263 patients: 71.66%), abdominal distension (341 patients: 92.92%), and nausea and vomiting (91 patients: 24.8%) supplemented with a positive abdominal radiograph. These features in the history supplemented by a clinical examination particularly focusing on abdominal girth measurement and elicitation of tenderness, per rectal examination and auscultation together with observation of multiple air-fluid levels in the supine radiograph of abdomen were used in making the diagnosis of acute intestinal obstruction. Patients with features of obstruction who were ultimately categorized as having ileus based on history of medication intake, electrolyte abnormalities, and confirmed by a barium examination were excluded from the study. All patients had a supine abdominal radiograph, routine blood tests and were offered HIV status evaluation prior to admission with proper consent (two patients refused HIV testing). Detailed evaluation of the postoperative progress of the patients and any note of complications and the cause of mortality were made.

### Ethical consideration

Informed consent was not obtained from the patients as it was a retrospective study.

## RESULTS

### Incidence

Of 3717 patients admitted on particular days of the week over a 3 year period (May 2005-April 2008), acute intestinal obstruction was diagnosed in 367 patients (9.87%).

### Sex and age distribution

Acute intestinal obstruction was seen to affect mostly males (276 patients, 75.20%) than females (91 patients, 24.79%). The most common occurrence was in the age group 20-60 years (235 patients, 64.03%), followed by the group >60 years (98 patients, 26.7%), while the least common occurrence was in the age group <20 years (34 patients, 9.26%). The mean age of our patients was 41.27 years while the median age was 44 years [Table 1].

**Table 1:** Age distribution of patients with acute intestinal obstruction

Age range (years)	No. of patients
12-20	34 (M:23, F:11)
20-30	42 (M: 35, F:7)
30-40	56 (M:41, F:15)
40-50	89 (M:69, F:20)
50-60	48 (M:31, F:17)
60-70	72 (M:57, F:15)
70-80	21 (M:16, F:5)
>80	5 (M:4, F:1)

### Etiology

The causes of acute intestinal obstruction in our patient population are enumerated in Table 2. Of the 365 patients tested, overall 8 patients had a positive HIV-ELISA test and 6 had full blown AIDS.

**Table 2:** Etiology of acute intestinal obstruction in our series

Etiology	Number of patients	Percentage
Obstructed/strangulated hernia	132	35.96
Malignancy	61	16.62
Adhesions	57	15.53
Intestinal tuberculosis	52	14.17
Volvulus	23	6.26
Intussusception	8	2.18
Miscellaneous	34	9.26
Total	367	

### Management

Patients were either subjected to conservative management (79 patients, 21.5%) or to operative intervention (288 patients, 78.5%) [Table 3]. Criteria for subjecting patients to surgery included increasing pain and distension with gross abdominal tenderness, tachycardia, and other features of toxicity like fever and leukocytosis and failure of non-operative treatment. For patients with intestinal tuberculosis, there was a higher threshold for surgery and only patients who had increasing distension, tachycardia, and no response to conservative treatment even after 3-4 days of admission were subjected to surgery, apart from those who were toxic on admission and those with evidence of hollow viscus perforation (free gas in radiograph) or intra-abdominal abscess formation. Surgery was performed within the first 24 h in the majority of patients (234, 81.25%), and the rest within the next 4 days. The selection criteria of the surgical procedure were based on the intraoperative findings. Obstructed hernia was managed by resection of the involved gut whenever gangrenous (29 cases) followed by anastomosis along with a primary repair of the hernia. Malignant disease was most common in the large bowel (58 cases) and was managed by primary resection in 46 cases, bypass in 8 cases, and stoma creation in 4 cases. Patients with malignancy were subjected to further treatment on an elective basis later on. Adhesions were managed by adhesiolysis and resection of the gut whenever gangrenous (16 cases). Intestinal TBC resulting in perforation was managed by resection of the gut with anastomosis or ileostomy. Volvulus was managed by primary resection and anastomosis (11 cases) or Hartmann’s procedure (8 cases).

**Table 3:** Intervention and outcome of patients with acute intestinal obstruction

Etiology	Management		Outcome		
	Conservative	Surgery	Complications	Death
Hernia	0	132	25	1
Neoplasm	9	52	21	3
Adhesions	34	23	5	1
Intestinal TB	26	26	23	14
Volvulus	4	19	5	1
Intussusception	0	8	4	2
Miscellaneous	6	28	12	5
Total	79	288	95	27

Surgery for intussusception was done in all cases (8 cases) and other conditions classed as miscellaneous were managed appropriately. Intestinal pseudo-obstruction resulting in a “negative” laparotomy occurred in seven cases. In these patients, contrast examination was not performed and a decision for laparotomy was taken based on history and clinical features.

### Morbidity

Complications in the postoperative period occurred in 95 patients (25.89%) [Table 3]. Of these, 38 patients (10.35%) had a single complication while the remaining 57 (15.54%) encountered more than one complication. Wound infection was the most common complication, occurring in 44 patients (11.99%), and of these, 21 patients (5.72%) required application of secondary sutures. Burst abdomen requiring emergency closure of abdomen occurred in 16 cases (4.4%). Prolonged ileus (more than 72 h) occurred in 34 patients (9.26%). Basal atelectasis was noted in 31 patients (8.45%) mostly of the elderly age group. Sepsis developed in seven patients with intestinal tuberculosis (13.46%). Small bowel fistula developed in seven patients (1.9%) and of them four (1.09%) required operative intervention in the long run.

### Mortality

In our case series, the mortality rate was 7.35% (27 patients). Of these, four patients died while on conservative management (5.06% of conservatively treated patients) and the rest following surgery. Two deaths (7.4% of deaths) occurred in the immediate postoperative period as a result of cardiac arrest following acute myocardial infarction. Five patients (18.52% of deaths) who developed sepsis in the postoperative period expired because of multi-organ failure. Mortality was high in the intestinal tuberculosis group (14 patients: 51.85% of total deaths) and all of them were grossly malnourished. Four patients had full blown AIDS (14.81% of deaths).

## DISCUSSION

Acute intestinal obstruction is one of the most common causes for surgical admissions worldwide. The etiology varies; however, adhesions[1] appear to be the most common cause in the Western world as well as in parts of Asia and Middle East.[2,3] However, in our study, obstructed hernia rather than adhesions appeared to be the most common cause followed by large bowel neoplasms as has been seen earlier in previous reports.[4,5] Intestinal tuberculosis also appeared to be an important factor in the etiology given the high prevalence of tuberculosis in the Indian subcontinent as well as the rising incidence of HIV in the Indian population.[6,7]

The gender discrepancy in our patients with males outnumbering females by a huge margin can be possibly accounted for, as a large number of our patients had obstructed inguinal hernia, and in our country we mostly have males who suffer from this condition. Also, women in rural India are mostly housewives which limit their exposure to tubercle bacilli in contrast to males. Also, volvulus and malignant disease of the gastrointestinal tract are more common in males as compared to females.

A critical factor in managing these patients is to determine whether patients can be subjected to conservative treatment or to emergency surgery.[8] Conservative therapy was typically advocated for patients with a preoperative diagnosis of band obstruction when the physiological parameters were within normal limits (keeping a low threshold for surgery) as also in patients with intestinal tuberculosis who were extremely malnourished and in frail patients who could not be subjected to surgery. Patients with band obstruction were diagnosed on the basis of history (recurrent bouts of intestinal obstruction managed conservatively earlier, history of laparotomy or appendicectomy in the past 2 years). However, when patients were followed up after the study duration, 45 of 52 patients with a diagnosis of band adhesions required surgery and the remaining were lost to follow up.

The mortality in our study seemed quite high compared to other similar studies, while the morbidity was comparable.[2,3]

There are several drawbacks in our study. Since our institution is a tertiary referral hospital, we mainly attended to cases which could not be managed under the primary or secondary level of health care; therefore, an accurate etiological assessment of acute intestinal obstruction might not have been reflected in our study. Also, most of our patients were from a poor socioeconomic status with a high prevalence of malnutrition; therefore, the morbidity and mortality are likely to be higher. The higher number of terminally ill patients as well as patients with AIDS also led to a higher mortality rate in our study.

## CONCLUSION

Despite the drawbacks in our study, it reveals that obstructed hernia rather than adhesions is the most common cause for acute intestinal obstruction in this part of India. Intestinal tuberculosis assumes great importance in this part of the country as a cause for obstruction. Lastly, this study highlights the importance of using universal precautions in the emergency setting because of the ever increasing number of HIV cases in the country.
